# Subclinical neurovascular and immune correlates of post-COVID-19 syndrome detected by retinal imaging

**DOI:** 10.1016/j.bbih.2026.101315

**Published:** 2026-07-25

**Authors:** Michael Wunderle, Rebecca Wicklein, Andrea Ribeiro, Javier Carbajo-Lozoya, Anna Wöhnl, Johanna Negele, Veronika Kesseler, Anna Wild, Samuel Niedermayer, Isabelle Lethen, Maciej Lech, Martin J. Menten, Linus Kreitner, Christoph Schmaderer, Timon Wallraven

**Affiliations:** a TUM School of Medicine and Health, Department of Nephrology, TUM University Hospital, Technical University of Munich, Munich, 81675, Germany; b TUM School of Medicine and Health, Department of Neurology, TUM University Hospital, Technical University of Munich, Munich, 81675, Germany; c Medizinische Klinik Und Poliklinik IV, LMU University Hospital Munich, Ziemssenstraße 5, Munich, 80336, Germany; d Chair for AI in Healthcare and Medicine, Technical University of Munich, Germany; e BioMedIA, Imperial College London, United Kingdom; f German Centre for Infection Research (DZIF), Munich Partner Site, Munich, Germany; g Site Lead, VADYS-ME Research Program, TUM University Hospital, Munich, Germany

**Keywords:** Post-COVID-19 syndrome, Optical coherence tomography (OCT), OCT angiography (OCTA), Retinal biomarkers, Neuroinflammation, Microvascular dysfunction

## Abstract

**Background:**

Post-COVID-19 Syndrome (PCS) encompasses a range of persistent symptoms, including cognitive and autonomic disturbances, potentially linked to microvascular or neuroinflammatory mechanisms. Retinal imaging provides a non-invasive window into the central nervous system and vascular integrity. This study aimed to assess retinal structural and microvascular alterations in PCS patients and explore their relationship with symptom severity.

**Methods:**

In this monocentric, cross-sectional, exploratory study, we performed Optical Coherence Tomography (OCT) and OCT Angiography (OCTA) in patients with PCS and a group of COVID-19 recovered individuals. Multiple circulating biomarkers reflecting endothelial dysfunction and chronic inflammation were measured. Associations with validated symptom scores (PCS Score, C19-YRS, PHQ-9, GAD-7) were evaluated.

**Results:**

After adjusting for age and gender, no significant differences were observed in OCTA-derived vessel density (VD) or foveolar avascular zone (FAZ) between PCS patients and COVID-19-recovered controls. However, PCS patients exhibited significant thinning of the peripapillary retinal nerve fibre layer (pRNFL), ganglion cell inner plexiform layer (GCIP), and total macular volume (TMV). Lower values of these structural parameters were consistently observed in patients with higher symptom severity. Vascular parameters (VD and FAZ) were not related to clinical scores. Higher MCP-1 levels, a chemokine associated with chronic inflammation, were linked to lower inner nuclear layer and GCIP thickness.

**Conclusion:**

Structural retinal alterations in PCS were associated with symptom burden and may reflect underlying neuroinflammatory or neurodegenerative processes. OCT parameters could serve as potential non-invasive biomarkers, warranting further investigation in longitudinal, multicenter studies.

## 1 Introduction

The coronavirus disease 2019 (COVID-19), caused by the severe acute respiratory syndrome coronavirus 2 (SARS-CoV-2), has led to significant global health consequences. Although initially recognized as a respiratory illness, it has become evident that COVID-19 also affects the vascular system, leading to endothelial dysfunction (ED) and ongoing inflammation with implications for various organs (Charfeddine et al., 2021; Kuchler et al., 2023a; Patrascu and Dumitru, 2025). A subset of patients continues to experience persistent symptoms after recovering from the acute infection, a condition referred to as Post-COVID-19 syndrome (PCS) (NICE, 2021) or Long COVID (Soriano et al., 2022). Given the typical symptoms of fatigue, dyspnea, autonomic dysfunction, neurocognitive impairment, psychiatric disorders, arthralgia, myalgia, exertional malaise, and more, there is considerable overlap between PCS and myalgic encephalomyelitis/chronic fatigue syndrome (ME/CFS), with some PCS patients meeting diagnostic criteria for ME/CFS, known as a sequela of other viral infections usually following pandemics (Michelen et al., 2021; Staub et al., 2024; Fark, 1991; Komaroff et al., 2023). Microvascular dysfunction has been proposed as a key factor in the development of these prolonged symptoms following SARS-CoV-2 infections (Kuodi et al., 2023; Hurme et al., 2025; Lowenstein et al., 2020; Mclaughlin et al., 2025), with potential consequences for the retinal vasculature (Schlick et al., 2022).

As a highly structured and accessible microvascular network, the retina provides a valuable model for investigating systemic vascular changes (Patton et al., 2005). The retina is an ideal site for studying local microcirculatory dynamics due to its complex mechanisms of blood flow autoregulation involving a tightly coordinated neurovascular unit (Kur et al., 2012). Optical coherence tomography angiography (OCTA) is a non-invasive imaging technique that enables detailed assessment of retinal capillary perfusion (Campbell et al., 2017). Previous studies have reported reduced vessel density following COVID-19, particularly in the superficial, intermediate, and deep capillary plexuses (Zapata et al., 2022; Banderas García et al., 2022; Turker et al., 2022; Abrishami et al., 2021; Guemes-Villahoz et al., 2021; Karkhur et al., 2023). Additional findings include enlargement of the foveal avascular zone (FAZ), reduced foveal vascular density (VD), and reduced central macular thickness (CMT = retinal thickness measured at macula) suggesting persistent vascular damage. Conversely, transient thickening of the retinal nerve fiber layer (RNFL) has been interpreted as an early inflammatory response rather than permanent axonal loss (Ozturk et al., 2025). Some of these changes were associated with the severity of the acute infection as well as with PCS symptom severity, indicating a potential role of SARS-CoV-2 in inducing long-term endothelial dysfunction (Schlick et al., 2022; Talebnejad et al., 2025). Additional evidence of microcirculatory impairment in COVID-19 has been provided by other investigations, including a comprehensive review by Ackermann et al., which delineates widespread endothelial injury and microvascular dysfunction across multiple organ systems (Davis et al., 2023).

In a previous study by our group using static and dynamic retinal vessel analysis (RVA), we identified both structural and functional vascular abnormalities in PCS patients, compared to SARS-CoV-2 naïve participants (Kuchler et al., 2023a). According to our findings, Invernizzi et al. described dilated retinal veins in COVID-19 patients (Invernizzi et al., 2021). These findings support the hypothesis of an altered retinal microvasculature in PCS. Building on this observation, we aimed to investigate whether similar microvascular alterations can be detected using OCT and OCTA.

While some studies have demonstrated correlations between the severity of acute SARS-CoV-2 infection and changes in OCTA parameters such as VD, no studies to date have established a clear relationship between persistent symptom burden in PCS and alterations in the retinal microcirculation (Hohberger et al., 2021). Therefore, it is still controversial whether the chronic changes seen in OCTA are a long-term consequence of the acute infection (e.g. microclotting, hypoxemia) or reflect an ongoing process of inflammatory or autoimmune genesis (Invernizzi et al., 2021; Noor et al., 2023).

To investigate whether persistent retinal changes in PCS are linked to systemic pathophysiology, we assessed circulating markers of endothelial dysfunction and chronic inflammation, which are frequently elevated in PCS and may contribute to microvascular impairment (Xu et al., 2023). In addition, we examined immunoglobulin G (IgG) subclasses, given their established roles in antiviral defense and autoimmune processes. Subclass-specific mechanisms - such as complement activation by IgG1 - have been implicated in neurovascular damage in diseases like AQP4-neuromyelitis-optica-spectrum-disease and systemic lupus erythematosus (Cho et al., 2024). These observations prompted us to explore whether similar humoral immune patterns might be reflected in retinal alterations in PCS.

While therapeutic strategies are currently being explored (Stein et al., 2025; España-Cueto et al., 2025), it remains unclear whether the severity of PCS symptoms can be objectively assessed using retinal microcirculation parameters derived from OCTA.

To address these knowledge gaps, we conducted a study using OCT and OCTA to analyze the retinal microcirculation and neuroretinal structure in patients with PCS and individuals who had recovered from COVID-19. As described in the “All eyes on PCS” study protocol (Kuchler et al., 2023b), we hypothesized that PCS patients may exhibit alterations in retinal vessel density within the superficial and deep vascular plexuses. Analyses of structural OCT parameters and their associations with clinical and laboratory markers were performed in an exploratory, hypothesis-generating manner. Characterizing these changes contributes to a better understanding of PCS pathophysiology and supports the evaluation of OCT and OCTA as non-invasive diagnostic and monitoring tools in post-viral syndromes.

## 2 Materials and methods

### 2.1 Study design and cohort

This manuscript is based on the “All Eyes on PCS study”, a prospective, observational, single-center investigation aimed at analyzing the retinal microvasculature in individuals with PCS while also conducting a thorough clinical assessment of the participants. The present analysis focused on OCTA-derived macular vascular parameters to characterize retinal microcirculation. Analyses of structural OCT parameters and their associations with clinical and laboratory markers were conducted in an exploratory, hypothesis-generating manner. The study is registered on ClinicalTrials.gov (https://clinicaltrials.gov/ct2/show/NCT05635552).

Between October 2022 and September 2023, a total of 105 PCS patients were enrolled. The majority (72.4%, 76/105) were recruited via social media, while the remaining 27.6% (29/105) were referred from the PCS outpatient clinic. Three participants were excluded from the final analysis due to specific reasons: one lacked sufficient evidence of a prior SARS-CoV-2 infection, another had no clear temporal association between infection and the onset of PCS symptoms, and the third did not exhibit PCS-related symptoms at the time of recruitment. OCT and OCTA data were obtained from 80 to 61 patients, respectively. The control group included 102 individuals who had fully recovered from SARS-CoV-2 infection and did not fulfill diagnostic criteria for PCS, with OCT and OCTA data available for 48 and 33 of them, respectively. In total, 82 OCTA scans from 52 participants were excluded due to insufficient image quality. Additional data loss resulted from participant refusal to undergo imaging. Further details on patient recruitment and clinical assessment are available in the previously published study protocol (Kuchler et al., 2023b).

### 2.2 Clinical assessment

To ensure a comprehensive evaluation, data collection was conducted through a standardized questionnaire and patient-reported outcome measures (PROMs). The questionnaire gathered demographic information, pre-existing medical conditions, current medications, and PCS symptoms, with a focus on 12 symptom complexes (Kuchler et al., 2023a). Additionally, PROMs were used to assess various aspects of PCS severity and patient well-being:

**PCS Severity:** Evaluated using the PCS Severity Score (Bahmer et al., 2022) and the COVID-19 Yorkshire Rehabilitation Scale (C19-YRS) (O'Connor et al., 2022).

**Fatigue:** Measured with the Fatigue Severity Scale (FSS) (Krupp et al., 1988).

**Depression:** Assessed using the Patient Health Questionnaire-9 (PHQ-9) (Costantini et al., 2021).

**Health-Related Quality of Life:** Determined through the EuroQol 5-Dimensions 5-Levels questionnaire (EQ (5DL)) (Herdman et al., 2011).

**ME/CFS Status:** Identified based on the Canadian Consensus Criteria (Encephalomyelitis/Chronic et al., 2015).

### 2.3 Laboratory measurements

Blood samples were collected, and standard laboratory measurements were analyzed following previously described protocols (Kuchler et al., 2023a). Specific biomarkers were assessed as follows:

**Cytokines and Chemokines:** Interleukin-6 (IL-6), Interleukin-8 (IL-8), Interferon gamma-induced protein 10 (IP10) and Monocyte Chemoattractant Protein-1 (MCP-1) were quantified using the Cytometric Bead Array Flex system (BD Biosciences, San Diego, US). Serum was used for these assessments. These biomarkers were selected based on their established roles in systemic inflammation (IL-6, IL-8), immune activation (IP-10), and monocyte recruitment and chronic inflammatory processes (MCP-1), which have been implicated in the pathophysiology of PCS (Almulla et al., 2024).

**Adhesion Molecules:** Intercellular Adhesion Molecule 1 (ICAM-1) and Vascular Cell Adhesion Molecule 1 (VCAM-1) were quantified using the Cytometric Bead Array Flex system (BD Biosciences, San Diego, US).

**Von Willebrand Factor (vWF:Ag) Levels:** Measured using the LIAPHEN™ vWF:Ag kit (Hyphen Biomed, Neuville-sur-Oise, France) through an immunoturbidimetric assay, following the manufacturer's guidelines in patient plasma.

### 2.4 OCT and OCTA assessments

Optical coherence tomography (OCT) and optical coherence tomography angiography (OCTA) were performed using a spectral-domain OCT device (Spectralis OCT2, Heidelberg Engineering, Germany), including its angiography module for OCTA acquisition. Macular OCT scans (30° × 25°) were acquired, and segmentation of retinal layers was performed using the vendor-provided software (Eye Explorer, v2.5.4), with manual correction when necessary. Image quality was assessed following the OSCAR-IB criteria (Tewarie et al., 2012).

En-face OCTA images were recorded centered on the fovea centralis (2.9 × 2.9 mm). To minimize motion artifacts, active eye tracking was employed by a software built-in algorithm. OCTA image quality was ensured using the OSCAR-MP criteria (Wicklein et al., 2023). Vessel densities in the superficial and deep vascular complexes (SVC and DVC) were analyzed around the fovea in 2.5 mm eccentricity (area 4.9 mm^2^) using a deep-learning-based segmentation approach as previously described (Kreitner et al., 2023), enabling automated extraction of the retinal vascular network and differentiation of vessel diameters.

The following retinal parameters were extracted:

### 2.5 Structural parameters

**All macular layer thickness parameters were derived from macular OCT scans**, unless otherwise specified**.**

**Inner Nuclear Layer thickness (INL, μm):** The retinal layer containing bipolar, horizontal, and amacrine cells, critical for signal transmission between photoreceptors and ganglion cells.

**Ganglion Cell Inner Plexiform Layer thickness (GCIP, μm):** A combined measure of the ganglion cell layer (GCL) and inner plexiform layer (IPL).

**Retinal Nerve Fiber Layer thickness (RNFL, μm):** Macular RNFL thickness. This layer contains axons of the ganglion cells, forming the optic nerve.

**Peripapillary Retinal Nerve Fiber Layer thickness (pRNFL, μm):** RNFL thickness measured in the peripapillary region, using a separate optic disc-centered scan.

**Total Macular Volume (TMV, mm^3^):** A volumetric measure of the macular region, providing insights into overall retinal structural integrity.

**Retinal Thickness (μm):** The total thickness of retinal layers at the center of fovea.

### 2.6 Vascular parameters

All vascular parameters were derived from OCTA scans centered on the macula.

We performed localized SVC and DVC analyses using the Early Treatment of Diabetic Retinopathy Study (ETDRS) grid (Early Treatment Diabetic Retinopathy Study, 1991). To specifically distinguish the retinal capillary network from larger vessel structures, vessel densities were categorized into diameters of <10 μm and >10 μm. The foveal avascular zone (FAZ) was segmented based on the SVC.

**Superficial Vascular Complex (SVC, %):** Defined as the region between the inner limiting membrane (ILM) and the inner plexiform layer (IPL). This layer primarily supplies the ganglion cell layer and the IPL.

**Deep Vascular Complex (DVC, %):** Defined as the region between IPL and the outer plexiform layer (OPL). This layer is responsible for supplying the deeper retinal structures.

**Foveal Avascular Zone (FAZ, mm^2^):** The capillary-free central region of the fovea, crucial for high-acuity vision. Changes in FAZ morphology are associated with microvascular dysfunction.

### 2.7 Statistical methodology

All statistical analyses were conducted using RStudio (Version 12.0, 2024). Normality was assessed using the Shapiro-Wilk test for smaller sample sizes, while for larger datasets (n > 100), the Kolmogorov-Smirnov test was applied. Normally distributed variables were summarized using the mean and standard deviation (SD), whereas non-normally distributed data were described with the median and interquartile range (IQR). Retinal parameters were not analyzed independently for each eye. Instead, a paired-eye statistical approach was used: if both eyes were available within the same analysis group, their mean was used as one data point, otherwise the available eye was included. Missing values were removed before analysis and visualization to ensure accuracy, either during data preprocessing or by enabling the na.rm = TRUE function in relevant computations.

Appropriate statistical tests were used based on the distribution of the data. For group comparisons, t-tests, Chi-Square tests, or the Mann-Whitney *U* test were applied. Ordinal variables were analyzed using proportional odds logistic regression (POLR), adjusting for age and gender. Additionally, age- and gender-adjusted p-values for continuous and binary baseline variables were obtained using multivariable linear and logistic regression models, respectively. As a sensitivity analysis, propensity score matching (1:1 nearest-neighbor matching with a caliper of 0.4 based on age and sex) was applied to reduce residual confounding between PCS patients and COVID-19-recovered controls. Correlation analyses were performed using Pearson's correlation for normally distributed data and Spearman's correlation for non-normally distributed variables. Receiver operating characteristic (ROC) analysis was used to assess the discriminative power of different variables. To ensure consistency between compared models and the corresponding AUC estimates, ROC analyses were performed using a complete-case approach, including only participants with complete data for all predictors entered into the respective model. In addition, univariate and multivariate logistic (for binary dependent variables) and linear regression models (for continuous dependent variables) were used to explore associations between variables and outcomes, adjusted for age and gender. Extreme outliers were identified using the interquartile range (IQR) method (values outside 1.5 × IQR) and subsequently excluded from further analyses.

## 3 Results

### 3.1 Baseline demographic data

The demographic and clinical characteristics of the two cohorts – individuals recovered from COVID-19 and patients with PCS – are summarized in detail in Table 1. A total of 80 PCS patients and 48 recovered controls were included in the analysis. Compared to COVID-19 recovered individuals, PCS patients were older (median age 40.0 vs. 26.5 years, p < 0.001), had a higher body mass index (BMI) (23.7 vs. 22.1 kg/m^2^, p = 0.029), and more frequently exhibited arterial hypertension (17.5% vs. 2.1%, p = 0.009) as well as hypercholesterolemia (48.7% vs. 21.4%, p = 0.006). However, after adjustment for age and sex, these differences in cardiovascular risk factors were no longer statistically significant.

**Table 1:** Baseline demographic characteristics of the two cohorts (COVID-19 recovered individuals and PCS patients). Unadjusted p-values reflect group comparisons using appropriate statistical tests; adjusted p-values account for age and gender.

	COVID-19 recovered N = 48	PCS patients N = 80	p-value	Adjusted p-value*
**Age in years (Median (IQR))**	26.5 (24.0 - 34.2)	40.0 (32.0 - 51.0)	<0.001	-
**Gender, male (n, (%))**	11 (22.9%)	20 (25.0%)	0.63	-
**BMI in kg/m^2^ (Median (IQR))**	22.1 (21.1 - 24.2)	23.7 (21.2 - 26.7)	0.03	0.40
**Obesity (n, (%))**	1 (2.1%)	11 (13.8%)	0.03	0.25
**Nicotine abuse (n, (%))**	5 (10.4%)	9 (11.2%)	1.00	0.69
**Arterial hypertension (n, %))**	1 (2.1%)	14 (17.5%)	0.009	0.15
**Diabetes mellitus II (n, (%))**	0 (0.0%)	1 (1.2%)	1.00	1.00
**Hypercholesterolemia (n, (%))**	9 (21.4%)	38 (48.7%)	0.006	0.08
**Severity of acute infection (WHO progression scale)**			<0.001	<0.001
**0**	1 (2.1%)	0 (0.0%)		
**1**	2 (4.2%)	1 (1.2%)		
**2**	43 (89.6%)	47 (58.8%)		
**3**	1 (2.1%)	28 (35.0%)		
**4**	0 (0.0%)	2 (2.5%)		
**5**	0 (0.0%)	1 (1.2%)		
**6**	0 (0.0%)	1 (1.2%)		
**8**	1 (2.1%)	0 (0.0%)		
**Variance**			0.003	0.003
**Unknown**	34 (72.3%)	45 (56.2%)		
**Alpha**	0 (0.0%)	7 (8.8%)		
**Delta**	0 (0.0%)	13 (16.2%)		
**Omicron**	13 (27.7%)	15 (18.8%)		
**Number of infections**			0.10	0.28
**1**	29 (60.4%)	53 (66.2%)		
**2**	14 (29.2%)	25 (31.2%)		
**3**	5 (10.4%)	1 (1.2%)		
**4**	0 (0.0%)	1 (1.2%)		
**Time since COVID-19 infection**				
**Mean (±SD)**	503.0 (±332.9)	476.4 (±272.3)	0.64	0.01
**Number of vaccinations**			0.001	<0.001
**0**	0 (0.0%)	5 (6.2%)		
**1**	1 (2.1%)	2 (2.5%)		
**2**	2 (4.3%)	21 (26.2%)		
**3**	30 (63.8%)	43 (53.8%)		
**4**	14 (29.8%)	7 (8.8%)		
**5**	0 (0.0%)	2 (2.5%)		
**Vaccination before infection (n, (%))**	43 (93.5%)	52 (67.5%)	0.002	0.65
**PCS duration**				
**Median (IQR)**	0.0 (0.0 - 0.0)	419.0 (301.0 - 603.0)	<0.001	<0.001
**Job loss (n, (%))**	0 (0.0%)	14 (17.9%)	0.005	0.99
**Sick leave (n, (%))**	0 (0.0%)	65 (81.2%)	<0.001	0.99
**Sick note (Median (IQR))**	0.0 (0.0 - 0.0)	200.0 (60.5 - 381.8)	<0.001	<0.001
**ME/CFS (n, (%))**	0 (0.0%)	53 (67.1%)	<0.001	<0.001
**C19-YRS (Mean (±SD))**	-	33.9 (±13.7)	-	-
**GAD7 (Median (IQR))**	-	5.0 (3.0 - 9.0)	-	-
**PHQ9 (Mean (±SD))**	-	10.8 (±4.3)	-	-
**Fatigue Severity Scale (Median (IQR))**	2.1 (1.6 - 2.9)	6.2 (5.3 - 6.7)	<0.001	<0.001
**PCS Severity Score (Mean (±SD))**	0.0 (±0.0)	37.5 (±10.2)	<0.001	<0.001
**Fatigue (n, (%))**	0 (0.0%)	77 (96.2%)	<0.001	<0.001
**Exercise intolerance (n, (%))**	1 (2.1%)	76 (95.0%)	<0.001	<0.001
**Brainfog (n, (%))**	0 (0.0%)	74 (92.5%)	<0.001	<0.001

**Fig. 1:**
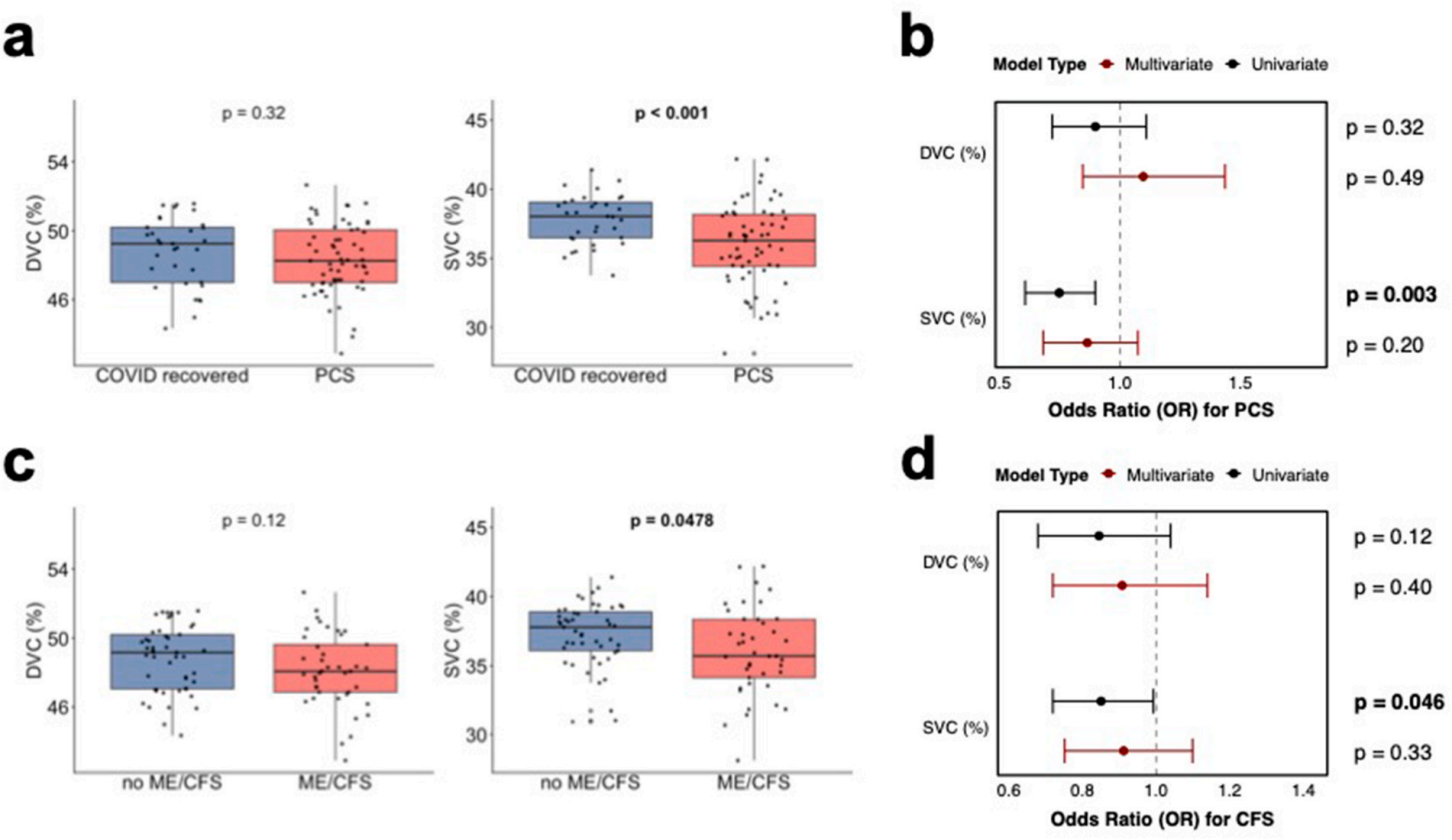
Boxplots show cohort differences between PCS and recovered participants for DVC and SVC **(a)**. Forest plot showing univariate and multivariate logistic regression analyses for DVC and SVC, adjusted for age and sex, with PCS as the dependent variable **(b)**. Boxplots show differences in vessel density between PCS patients with and without ME/CFS **(c)**. Forest plot showing univariate and multivariate logistic regression analyses for DVC and SVC, adjusted for age and sex, with ME/CFS as the dependent variable **(d)**.

To assess retinal microvascular alterations in patients with PCS, we analyzed vessel densities within the superficial (SVC) and deep vascular complex (DVC). As shown in Fig. 1a, PCS patients exhibited significantly reduced vessel densities in the SVC compared to COVID-19 recovered controls, while DVC parameters were less affected. To evaluate the diagnostic relevance of these microvascular changes, logistic regression analyses were performed (Fig. 1b). In univariate models, reduced SVC vessel density was significantly associated with PCS status. However, after adjustment for age and gender, this association was no longer statistically significant.

In a subgroup analysis of PCS patients with and without comorbid ME/CFS (Fig. 1c), we again observed lower SVC vessel densities in patients with ME/CFS. In multivariate regression models (Fig. 1d), this association was no longer statistically significant after adjustment for age and sex.

To investigate whether retinal microvascular alterations in PCS patients are associated with subjective symptom burden, we performed exploratory correlation analyses between vessel density and patient-reported outcome measures (PROMs), including the PCS Severity Score, C19-YRS, PHQ-9, and GAD-7. While a weak negative trend was noted between the SVC and the C19-YRS as well as the PHQ-9, none of the associations reached statistical significance (see Supplementary Figure 1). Likewise, vessel densities in the DVC showed no associations with symptom severity.

To evaluate potential macular perfusion deficits in PCS, we also assessed the size of the foveal avascular zone (FAZ). As shown in Supplementary Figure 2, FAZ area did not differ significantly between PCS patients and recovered controls, nor between PCS patients with and without comorbid ME/CFS. Furthermore, no relevant correlations were observed between FAZ area and PROMs. These results indicate that central macular perfusion, as reflected by FAZ size, is preserved in PCS, even in individuals with pronounced symptom burden.

As shown in Fig. 1 and Supplementary Fig. 1, retinal vessel density alone did not provide a robust distinction between PCS patients and recovered controls, nor did it correlate with subjective symptom burden. Nevertheless, given previous reports suggesting microvascular dysfunction and neuroinflammatory processes in PCS, we explored whether structural retinal changes might reflect disease-relevant tissue alterations not captured by vessel density alone.

**Fig. 2:**
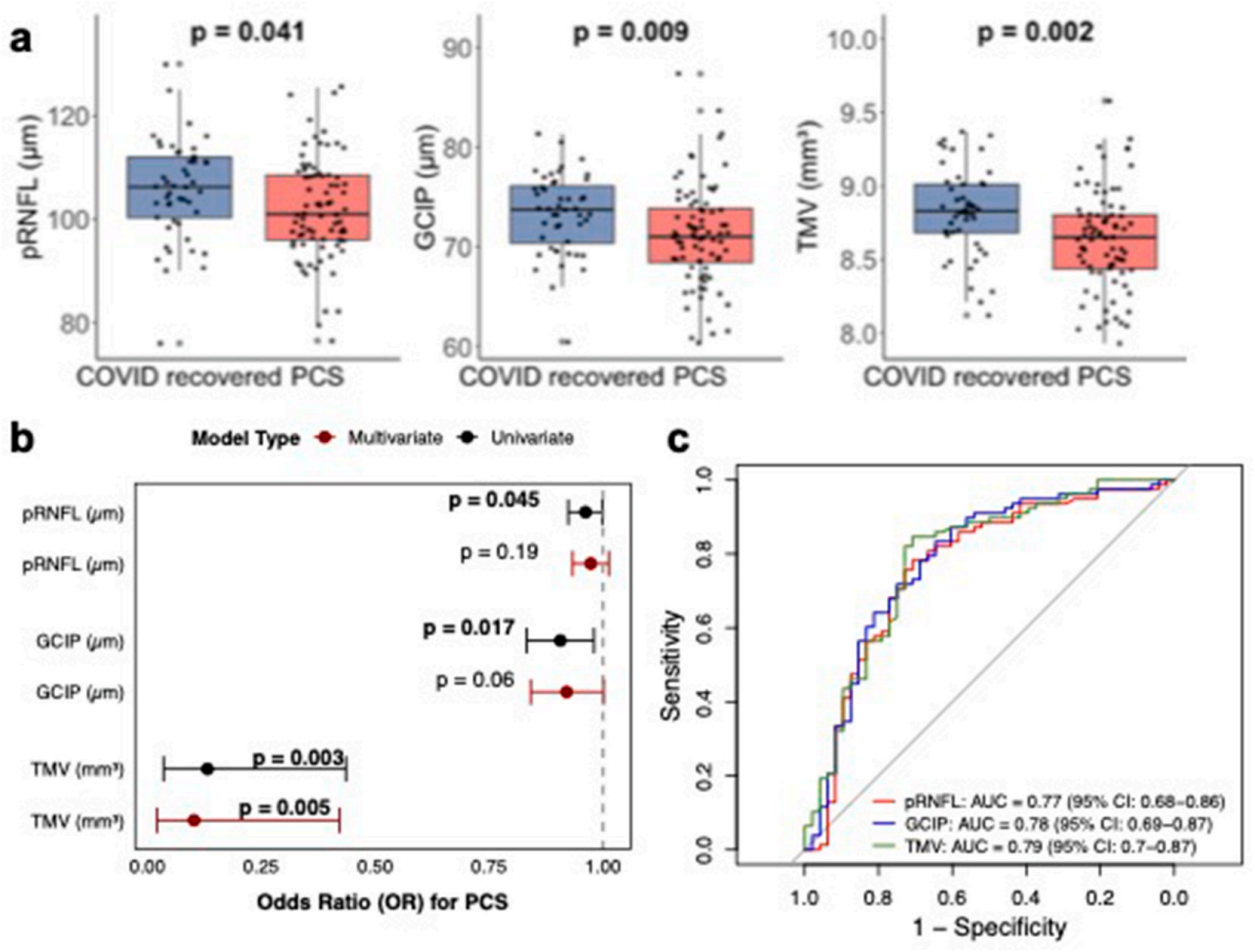
Panel **a** displays boxplots comparing OCT and OCTA parameters between patients with PCS and recovered controls. Statistical comparisons were performed using independent *t*-test. Panel **b** presents the results of both uni- and multivariate logistic regression models assessing the association between individual OCT parameters and the likelihood of having PCS, adjusted for age and gender. Regression coefficients are visualized alongside confidence intervals and p-values. Panel **c** shows receiver operating characteristic (ROC) curves adjusted for age and gender for each OCT parameter in predicting PCS status. Abbreviations: PCS = post-COVID syndrome; pRNFL = peripapillary retinal nerve fiber layer; GCIP = ganglion cell inner plexiform layer; TMV = total macular volume; SVC = superficial vascular complex; AUC = area under the curve; CI = confidence interval.

Exploratory analyses of structural OCT parameters are presented in Fig. 2a. PCS patients showed significant thinning of the peripapillary retinal nerve fiber layer (pRNFL), the ganglion cell-inner plexiform layer (GCIP), and a reduction in total macular volume (TMV), while inner nuclear layer (INL) and overall retinal thickness did not differ significantly (data not shown). In multivariate logistic regression models (Fig. 2b), TMV was significantly associated with PCS status, and the GCIP by trend was associated with PCS status. For transparency, all retinal structural and microvascular parameters, including non-significant findings, are summarized in Supplementary Tables 1 and 2

Exploratory receiver operating characteristic (ROC) curve analyses confirmed the diagnostic value of these structural parameters (Fig. 2c). The performance of individual OCT metrics was moderate overall, but improved after adjusting for potential confounders, such as age and sex. GCIP and TMV showed the highest classification accuracy among all tested variables. This was further supported by combined models shown in supplementary Figure 3, where integrating GCIP thickness with SVC, TMV, or pRNFL resulted in an additional gain in diagnostic performance. These results suggest that structural OCT measures – alone or in combination – may capture subtle disease-related changes in PCS.

To address potential residual confounding due to the marked age difference between PCS patients and COVID-19-recovered controls, we performed a propensity score-matched sensitivity analysis using 1:1 nearest-neighbor matching based on age and sex. After matching, the cohorts were well balanced with respect to demographic variables and major clinical characteristics (Supplementary Table 3). The overall pattern of results was consistent with the primary analyses, with structural OCT parameters showing nominal group differences, while OCTA-derived vessel density measures remained largely unchanged (Supplementary Tables 4 and 5).

**Fig. 3:**
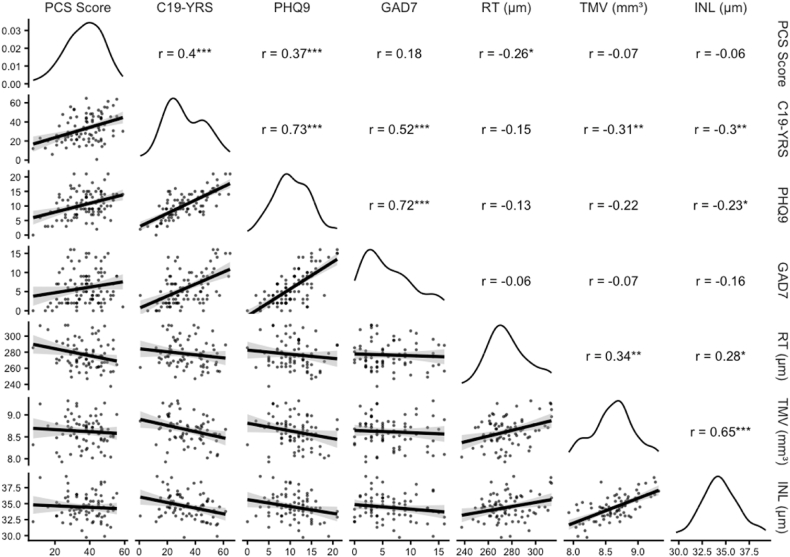
Pairwise correlation matrix including PROMs − PCS Severity Score, C19-Yorkshire Rehabilitation Scale Symptom Severity (C19-YRS), Patient Health Questionnaire-9 (PHQ-9), and Generalized Anxiety Disorder Scale-7 (GAD-7) − and OCT parameters: inner nuclear layer (INL), retinal thickness (RT) and total macular volume (TMV). The diagonal panels show the distribution of each variable as kernel density plots. Lower panels display scatterplots with fitted linear regression lines and 95% confidence intervals. Upper panels present Pearson correlation coefficients (R), with significance levels indicated as *p < 0.05, **p < 0.01, ***p < 0.001. Complete cases were used for all pairwise correlations.

To explore the association between patient-reported symptom burden and retinal structural parameters, we performed correlation analyses between clinical scores and both global and layer-specific retinal measures (Fig. 3). Correlation analyses with biomarkers and PROMs were conducted in an exploratory manner without formal correction for multiple testing. As in previous analyses, PROMs showed strong intercorrelations, particularly between C19-YRS, PHQ-9, and GAD-7 scores. Significant negative correlations were observed between INL thickness and C19-YRS (r = −0.30, p = 0.010) as well as PHQ-9 (r = −0.23, p = 0.043). Similarly, higher C19-YRS scores were associated with lower TMV (r = −0.31, p = 0.010. Lower RT was associated with higher PCS Severity Scores (r = −0.26, p = 0.021). These findings suggest a link between subjective symptom burden and subtle retinal thinning, particularly within inner retinal layers and macular volume. GCIP and pRNFL were included in the correlation analyses but are not shown in Fig. 3, as no significant associations with PROMs were observed.

Given the observed thinning of inner retinal layers in PCS patients, we next aimed to explore whether these structural alterations might be linked to systemic immune activity. Specifically, we investigated associations between retinal OCT parameters and serum Immunoglobulin G (IgG) subclass levels, as potential markers of immune system dysregulation.

**Fig. 4:**
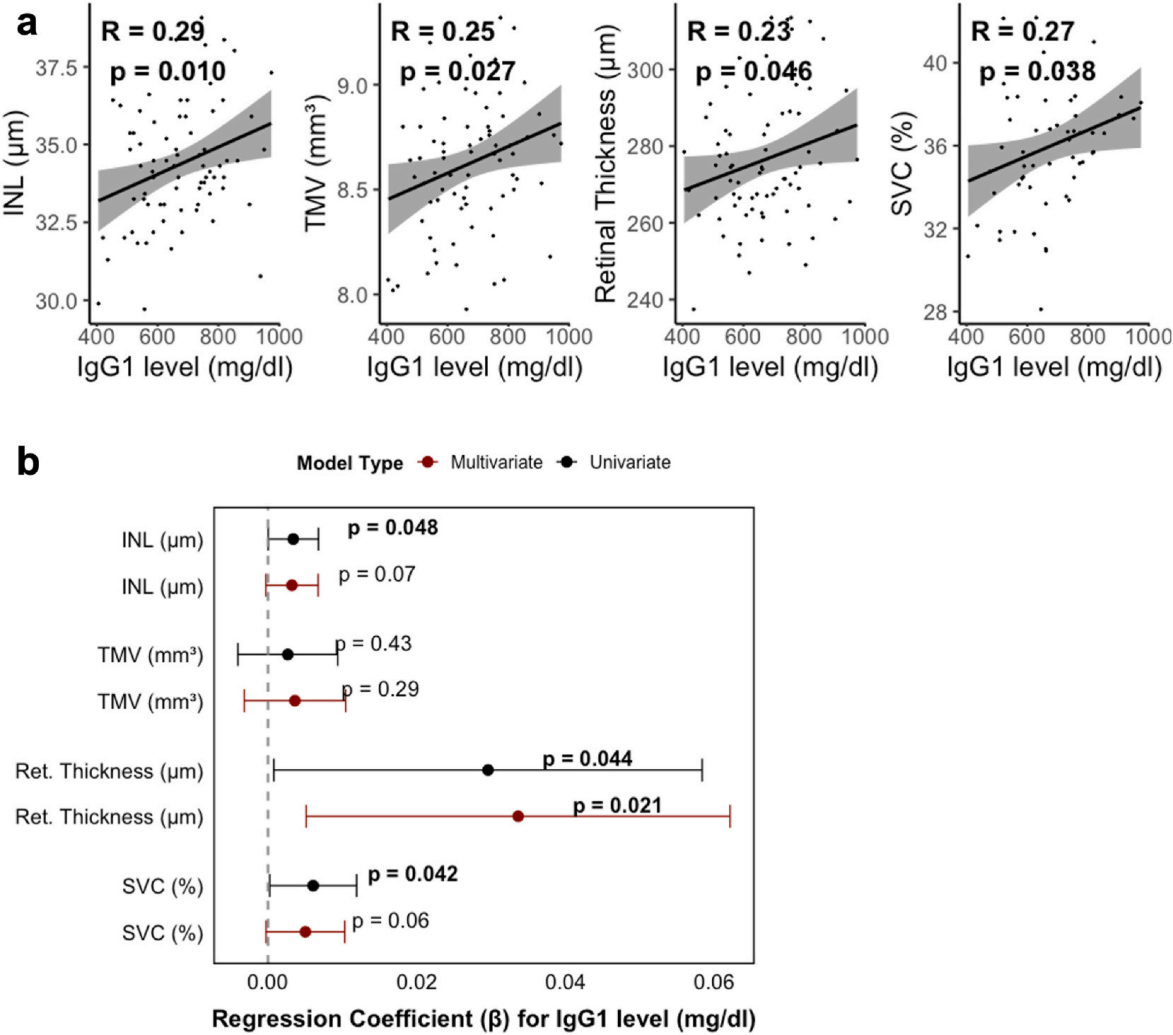
Scatterplots show significant correlations for various OCT and OCTA parameters and IgG1 levels **(a)**. Forest Plot shows uni- and multivariate linear regression analysis for INL, TMV, RT and SVC with IgG1 level as the dependent variable. Regression coefficients with corresponding confidence intervals and p-values are shown **(b)**.

As shown in Fig. 4a, IgG1 levels showed positive correlations with SVC, TMV, INL thickness, and overall retinal thickness. These relationships were further examined in uni- and multivariate linear regression models (Fig. 4b). In the multivariate analysis, the association between IgG1 and retinal thickness remained statistically significant, while associations with INL and SVC showed trends toward significance. This supports a potential link between systemic immune status and retinal integrity in PCS.

To assess the potential involvement of inflammatory and vascular mechanisms in retinal alterations in PCS, associations between OCT and OCTA parameters and circulating biomarkers of inflammation and ED were analyzed. This included cytokines, chemokines, adhesion molecules, and factors involved in vascular homeostasis.

**Fig. 5:**
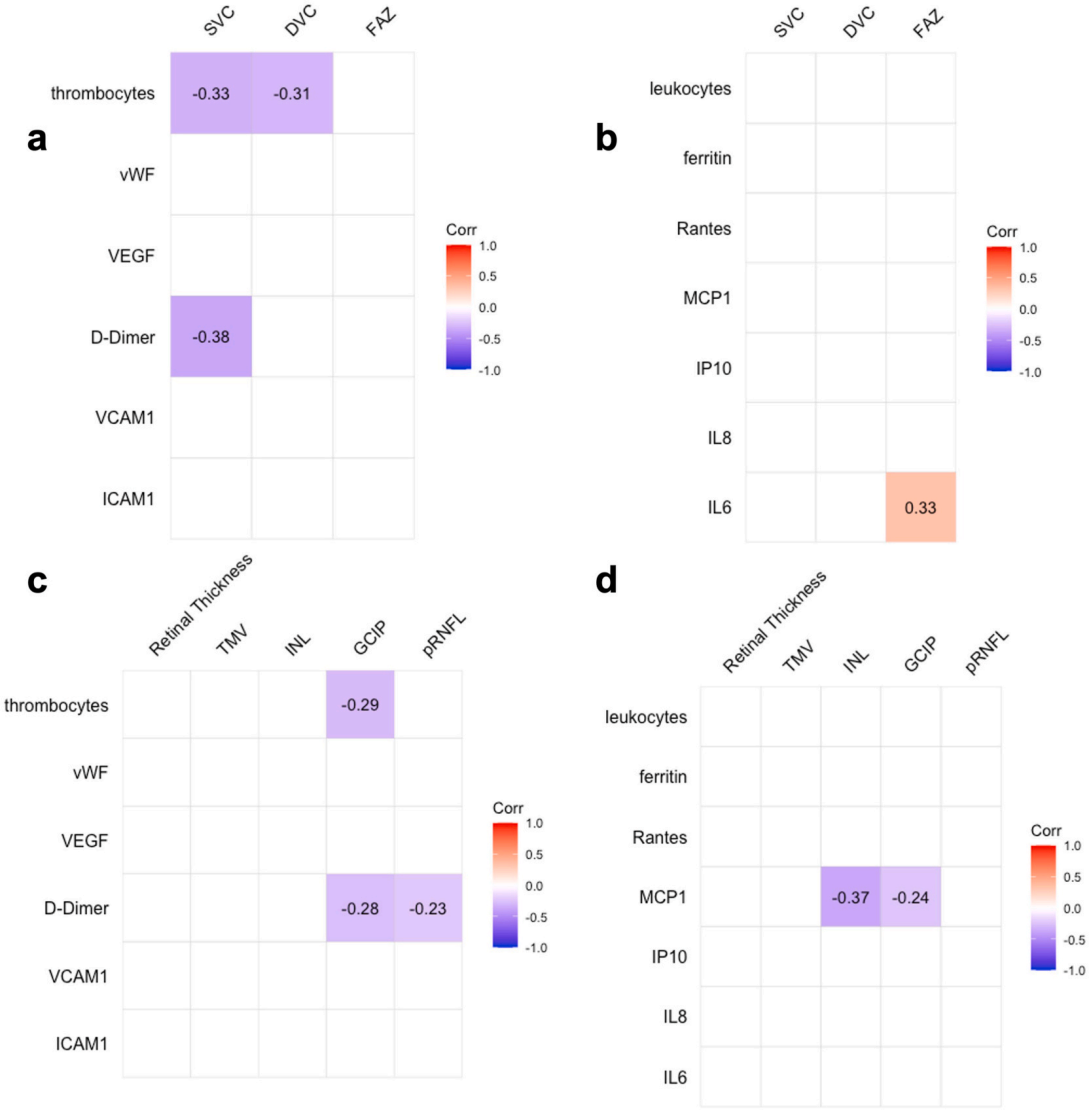
Correlation plots illustrate relationships between OCTA parameters with markers of endothelial dysfunction **(a)** and inflammation **(b)** and between OCT parameters between markers of endothelial dysfunction **(c)** and inflammation **(d)**. Correlations were calculated using Pearson's test for normally distributed variables and Spearman's test for non-normally distributed variables. Only statistically significant correlations (p < 0.05) are displayed with the corresponding r value. The color intensity of each tile reflects the strength of the correlation. (For interpretation of the references to color in this figure legend, the reader is referred to the Web version of this article.)

As shown in Fig. 5a and b, higher D-dimer levels were linked to lower SVC density. Higher D-dimer levels were also associated with lower GCIP and pRNFL thickness. In addition, higher thrombocyte counts were related to lower GCIP thickness. No significant correlations were found for other markers of ED.

Associations with markers of systemic inflammation are shown in Fig. 5c and d. Higher MCP-1 levels were associated with lower INL thickness (r = −0.37) and lower GCIP thickness (r = −0.24). No significant correlations were observed between structural OCT parameters and IP10, RANTES, or IL-8.

## 4 Discussion

This study investigated structural and vascular retinal changes in PCS patients using OCT and OCTA, comparing them with recovered controls. The primary goal was to assess differences in retinal parameters between PCS patients and fully recovered individuals. The secondary goal was to explore associations between retinal findings and both PROMs and circulating biomarkers indicative of chronic inflammation and endothelial dysfunction. Our results highlight subtle but consistent alterations in retinal layer thickness in PCS, with limited correlation to subjective symptom burden and markers of immune and endothelial dysregulation. These findings underscore the potential of OCT and OCTA as non-invasive biomarkers for assessing microvascular integrity in PCS patients.

### 4.1 Retinal imaging suggests vascular dysregulation rather than structural capillary damage in PCS

While OCTA has previously been applied in PCS populations, this is the first study to correlate OCTA-derived microvascular parameters with a range of validated PROMs and PCS-specific disease severity scores. Several studies have examined OCTA changes in post-COVID-19 populations (Banderas García et al., 2022; Cennamo et al., 2021; Bilbao-Malavé et al., 2021; Turker et al., 2021; González-Zamora et al., 2021), including some specifically focusing on PCS cohorts (Schlick et al., 2022; Szewczykowski et al., 2022). The results across studies are heterogeneous and sometimes contradictory. This may in part be explained by the frequently small sample sizes and differences in the timing and severity of infection, as well as inconsistent adjustment for potential confounding factors such as age, cardiovascular comorbidities, and smoking status (Jerratsch et al., 2024). Additionally, many studies use SARS-CoV-2–naïve individuals as control groups rather than individuals who have recovered from COVID-19, which may limit the specificity of observed differences to PCS rather than general post-infectious effects.

Several studies that examined patients in the early convalescent phase (weeks after infection) have reported significantly reduced VD in both SVC and DVC. Turker et al. (2021) and González-Zamora et al. (2021) found lower VD in both plexuses shortly after acute COVID-19. Similarly, mid-to long-term studies up to eight months post-infection also observed reductions in VD (Banderas García et al., 2022; Cennamo et al., 2021; Bilbao-Malavé et al., 2021), suggesting that retinal microvascular changes may persist beyond the acute phase. However, most of these studies examined general post-COVID-19 populations, including COVID-19 recovered patients, rather than well-characterized PCS cohorts. Furthermore, OCTA depicts blood flow rather than structure, so these observations could also be a result of vasoconstriction.

Multiple pathophysiological findings suggest impaired microcirculation in PCS. Kubánková et al. reported persistent deformation of red and white blood cells months after SARS-CoV-2 infection (Kubánková et al., 2021). Furthermore, autoantibodies against G-protein-coupled receptors (GPCR-AAbs) and β-adrenergic receptors have been detected in a substantial proportion of PCS patients (Sotzny et al., 2022; Wallukat et al., 2021). In a more targeted cohort, Szewczykowski et al. demonstrated significantly reduced VD in both the superficial and deep vascular complexes (SVC, DVC) in 40 PCS patients, all of whom were GPCR-AAbs positive (Szewczykowski et al., 2022). Similarly, Schlick et al. reported reduced VD in the intermediate capillary plexus (ICP) in 173 PCS patients compared with healthy controls (Schlick et al., 2022). These findings suggest the existence of distinct PCS subgroups – potentially driven by immune mechanisms – in which microvascular dysfunction is particularly pronounced. Such evidence underscores the heterogeneity of the PCS population, which must be considered when interpreting microvascular alterations in this context.

In contrast, our study did not reveal significant differences in VD between PCS patients and controls, nor any meaningful correlations between VD and PROMs such as the PCS Score, C19-YRS, PHQ-9, or GAD-7. This lack of association remained unchanged when stratified by vessel diameter. The foveal avascular zone (FAZ), a surrogate marker of central macular perfusion, was also preserved and showed no association with symptom burden or comorbid ME/CFS. These results suggest that, in our cohort, retinal microvascular perfusion, measured by OCTA, is not a reliable marker of subjective symptom severity in PCS. This divergence from prior OCTA studies reporting reduced retinal vessel density in PCS (Schlick et al., 2022; Szewczykowski et al., 2022) may reflect differences in cohort characteristics, including the longer time since infection, milder acute disease, and the use of COVID-19-recovered rather than SARS-CoV-2-naïve controls.

Our findings are consistent with those of Noor et al., who also reported no OCTA abnormalities or structural retinal changes in PCS patients with mild disease and a longer interval since infection. Rather than indicating full recovery of retinal microcirculation, these results may suggest that OCTA is not the most suitable tool for detecting microvascular alterations in this context. As OCTA is a metabolic imaging modality rather than a structural one, it does not reflect vascular changes as long as blood flow remains constant. Importantly, our previous investigations using retinal vessel analysis (RVA) in the same cohort revealed functional abnormalities, including reduced venous flicker-induced dilation (vFID) and changes in arteriovenous ratio (AVR) (Kuchler et al., 2023a). Unlike OCTA, which provides static structural information about capillary perfusion, RVA assesses dynamic vascular reactivity and larger vessel morphology in the peripapillary region. In our cohort, the functional assessment via RVA appeared more sensitive than OCTA, potentially due to its ability to capture autoregulatory dysfunction. The absence of OCTA-detectable abnormalities despite RVA-detected changes suggests that PCS-associated vascular dysregulation may preferentially affect larger vessels or dynamic regulatory mechanisms rather than resting capillary flow. The underlying reasons for this discrepancy remain unclear, but our findings highlight the importance of method selection when assessing retinal microcirculation in PCS.

Taken together, these results highlight key methodological and physiological distinctions between retinal imaging modalities in PCS. While OCTA remains a valuable tool for detecting microvascular alterations, it may not fully capture the complexity of vascular dysfunction in PCS, particularly in the absence of structural vessel damage. Multimodal vascular assessment including both static and dynamic imaging may provide a more comprehensive understanding of PCS-associated vascular pathology. These findings suggest that the absence of significant OCTA findings does not contradict the RVA results but rather suggests that microvascular perfusion may be relatively preserved at rest, despite functional impairments in vascular autoregulation. It also suggests that PCS-associated ED may preferentially affect larger-caliber vessels or dynamic vascular responsiveness, which OCTA alone may not adequately reflect. Future studies should incorporate longitudinal designs, standardized definitions of PCS, and stratification by immunological, autonomic, or severity-based subtypes to better define the role of retinal biomarkers in diagnosis, monitoring, and therapeutic decision-making in PCS.

### 4.2 Structural retinal alterations in PCS: associations with neuroretinal changes

Several studies have investigated retinal changes using OCT in post-COVID-19 patients, including some with PCS. Findings vary, with reports of both thinning and thickening in inner retinal layers such as the Retinal Nerve Fiber Layer (RNFL), Ganglion Cell Layer (GCL), and Inner Plexiform Layer (IPL), possibly reflecting neuroinflammatory or ischemic mechanisms (Jerratsch et al., 2024). However, only a few studies focus specifically on PCS cohorts, often with small sample sizes and inconsistent control for confounders. Importantly, variability in the time elapsed since acute infection further complicates interpretation.

Kanra et al. (2022) investigated 34 patients diagnosed with Long-COVID and reported significant thinning in at least half of the RNFL, GCL, IPL, and the overall inner retinal layer compared to healthy controls. No significant differences were found in the peripapillary RNFL (pRNFL) or central macular thickness. In contrast, Dağ Şeker et al. (Dağ Şeker et al., 2023) examined 27 recovered COVID-19 patients – using the term "Long-COVID" to describe general post-infectious sequelae rather than the clinically defined syndrome – and 27 healthy controls. They found thinning in segments of the macula as well as in the RNFL, pRNFL, GCIP and outer nuclear layer (ONL) at both one- and twelve-month post-infection.

In contrast, several studies have investigated retinal structural changes in the early period following SARS-CoV-2 infection, typically within the first few weeks. Oren et al. (2021) examined patients 14-30 days after symptom onset and reported increased central macular thickness (CMT) in the COVID-19 group, while the GCIP and INL were significantly thinner. No significant differences were observed in pRNFL thickness. Burgos-Blasco et al. (2022) found a significantly increased pRNFL thickness in recovered COVID-19 patients four weeks after infection. Meanwhile, González-Zamora (González-Zamora et al., 2021) et al. found no significant differences in retinal or RNFL thickness two weeks post-infection.

In our study, the median time since the acute infection was 476.4 (±272.3) days, which is much longer than in the studies mentioned above. PCS patients in our cohort showed significant thinning of the pRNFL, GCIP and TMV, while INL and total retinal thickness remained unchanged.

These findings may reflect the temporal dynamics of inflammation-related retinal changes following SARS-CoV-2 infection. In the early post-infectious phase, localized inflammation, particularly in the peripapillary region, may contribute to transient thickening of the RNFL, as observed by Burgos-Blasco et al. In contrast, studies examining patients weeks to months after infection, such as those by Kanra et al. and Dağ Şeker et al., more frequently report thinning of inner retinal layers, including the RNFL, pRNFL and GCIP. This suggests that persistent or unresolved inflammation in later stages may be associated with retinal thinning observed on OCT. The shift from inflammatory swelling to degenerative thinning may therefore underlie the divergent findings across timepoints.

Importantly, our observations of pRNFL and GCIP thinning in PCS patients are structurally similar to findings observed in neurodegenerative diseases. A recent meta-analysis in multiple sclerosis (MS) demonstrated that patients consistently exhibit significant thinning of both pRNFL and GCIP layers compared with healthy controls, reflecting axonal and neuronal loss as hallmarks of central nervous system degeneration (Filippatou et al., 2025). Beyond MS, similar OCT-derived retinal alterations have been reported in other autoimmune neuroinflammatory conditions such as neuromyelitis optica spectrum disorder (Oertel et al., 2021), as well as in systemic autoimmune diseases like systemic lupus erythematosus (Dias-Santos et al., 2021). Moreover, comparable retinal changes, including pRNFL and GCIP thinning, have also been described in neurodegenerative disorders such as Parkinson's disease or Huntington's disease (Biousse et al., 2022). These parallels might highlight similarities in retinal thinning patterns. However, whether comparable neurodegenerative mechanisms contribute to retinal alterations in PCS remain speculative.

In addition, several OCT-derived parameters, including pRNFL, GCIP and TMV, showed acceptable to good predictive value for detecting PCS, with age- and sex-adjusted AUCs ranging from 0.77 to 0.78.Higher PCS symptom burden was related to lower retinal thickness, as indicated by the PCS Score, with similar but no significant associations observed for C19-YRS and PHQ-9. In addition, lower INL thickness was associated with higher C19-YRS scores. These associations highlight the potential clinical relevance of retinal biomarkers for assessing symptom severity in PCS. The association of OCT parameters with both structural alterations and symptom burden suggests their potential relevance for understanding disease mechanisms and stratifying patient groups in future studies.

### 4.3 Retinal-IgG signature and possible implications in PCS pathophysiology

We identified a retinal-IgG signature in PCS patients, characterized by higher IgG1 levels being associated with greater retinal thickness, GCIP thickness, and SVC density, while higher IgG1 levels were associated with smaller FAZ area. Notably, retinal thickness maintained a robust association with IgG1 after adjusting for demographic and clinical confounders (Fig. 4), showing a thinner retina in patients with lower IgG1 levels.

These findings align in part with existing knowledge about IgG1 and IgG3, subclasses crucial in antiviral immune responses, as previously demonstrated in Epstein–Barr virus infections (Karsten et al., 2022) and hepatitis C, where elevated levels reflect effective antiviral activity (Ye et al., 2024). Moreover, IgG1 subclasses can induce complement-dependent cytotoxicity, relevant in autoimmune demyelinating disorders (Masciocchi et al., 2025). Chronic inflammatory or autoimmune conditions might lead to continuous Fcγ receptor-mediated endocytosis of IgG1-antigen complexes, resulting in enhanced internalization, subsequent degradation, and potential alterations in circulating IgG1 concentrations (Early Treatment Diabetic Retinopathy Study, 1991).

Collectively, our results suggest that retinal structural and vascular alterations in PCS could be linked to persistent systemic immune activation or inflammation, reflected by altered IgG1 dynamics. Lower circulating IgG1 levels, associated with reduced retinal thickness, may indicate ongoing inflammation and immune dysregulation in PCS, although such associations may also be influenced by broader systemic immune status or residual clinical and demographic confounding. Specifically, decreased IgG1 could reflect enhanced immune activation or altered immunoglobulin dynamics, while a direct contribution to retinal structural changes remains hypothetical. Alternatively, lowered IgG1 might represent impaired immune function and incomplete viral clearance, maintaining persistent inflammation. Further studies are needed to elucidate the exact role of IgG1 and its involvement in complement-mediated mechanisms or persistent viral activity within the pathogenesis of PCS.

### 4.4 Retinal alterations and markers of endothelial dysfunction and inflammation in PCS

Our findings highlight significant correlations between structural retinal parameters detectable via OCT and systemic markers of ED and inflammation in PCS patients, which may provide insights into the underlying pathophysiology of PCS. We observed associations between various retinal layer parameters with endothelial markers such as thrombocytes, and D-dimer, as well as the inflammatory marker MCP-1 (Fig. 5).

These correlations align with previously described mechanisms of chronic endothelialitis, disrupted nitric oxide signaling, increased vascular permeability, and pro-thrombotic conditions characteristic of PCS (Barrett et al., 2021; Halawa et al., 2022; Varga et al., 2020; Pandian et al., 2025; Santoro et al., 2023).

Such persistent endothelial and inflammatory processes may be associated with systemic and neurovascular alterations, mirrored by structural retinal changes detectable via OCT and OCTA imaging. Our data thus supports the concept of retinal imaging as a valuable tool for assessing systemic microvascular and inflammatory involvement in PCS patients.

Moreover, these retinal changes may reflect deeper alterations in neurovascular coupling, the dynamic interaction between neuronal activity, glial support, and microvascular perfusion (Kempuraj et al., 2024). Neuroinflammation in PCS, marked by sustained microglial activation and cytokine dysregulation, is thought to compromise this coupling, potentially leading to cerebral hypoperfusion and neural injury (Vlaicu et al., 2023; Chaves-Filho et al., 2023; CJ and AS, 2025). Recent evidence from individuals with PCS and cognitive impairment demonstrates that persistent systemic inflammation and blood-brain barrier (BBB) disruption are central features of the condition. Specifically, patients with brain fog exhibited increased BBB permeability in frontal and temporal brain regions alongside elevated serum levels of S100β and TGFβ, markers associated with astrocytic activation and vascular damage (Greene et al., 2024). Given the anatomical and physiological parallels between the retina and the brain, such dysregulation may manifest in the retina as structural changes, such as thinning of the inner retinal layers. These alterations are readily captured by OCT, supporting its potential as a non-invasive modality to investigate the systemic and cerebral consequences of PCS. In our study, we primarily observed alterations in retinal layer thickness, while microvascular OCTA parameters remained largely unaltered. This suggests that in the context of PCS, structural neuroretinal changes may be more prominent or more readily detectable than microvascular alterations.

### 4.5 Strengths and limitations

This study has several notable strengths that contribute to the growing body of literature on PCS and retinal imaging. To our knowledge, it is the first to systematically correlate OCTA-derived microvascular parameters, including vessel densities in the superficial and deep vascular complexes, with a range of validated patient-reported outcome measures (PROMs) and clinical disease severity scores in PCS. Our PCS cohort is well-characterized, with inclusion based on current consensus definitions and standardized symptom assessments, enhancing comparability and interpretability. Importantly, the median time since infection exceeded 15 months, allowing us to assess long-term retinal changes well beyond the acute or subacute stages. Notably, we identified structural retinal alterations (e.g., thinning of pRNFL, GCIP, and TMV) that were significantly associated with symptom severity, suggesting potential utility of OCT-based metrics as objective, non-invasive biomarkers in PCS. These findings may inform future studies on patient stratification and therapeutic monitoring. Finally, the study design accounted for potential confounding variables and included careful interpretation of null findings, ensuring methodological transparency and robustness of conclusions.

However, certain limitations must be acknowledged. This was a monocentric study, which may limit the generalizability of our findings. A notable number of OCTA scans were excluded due to participant refusal or insufficient image quality, which may have introduced selection bias and could have affected the representativeness of the analyzed cohort and the detection of subtle microvascular alterations. Although we adjusted for age differences between PCS patients and controls using multivariate models, some residual confounding cannot be excluded. To further address this limitation, we performed a propensity score-matched sensitivity analysis based on age and sex, which yielded results consistent with the primary analysis. The cross-sectional design limits conclusions about the temporal progression or persistence of retinal changes in PCS. In addition, the parent study was originally designed to investigate retinal microcirculation, whereas structural OCT analyses and retinal associations with clinical and laboratory markers represent exploratory extensions of this protocol-defined framework. Accordingly, these findings should be interpreted as hypothesis-generating rather than confirmatory. Importantly, OCT-derived retinal thinning cannot be directly interpreted as evidence of neurodegeneration but rather reflects unspecific structural alterations that may result from a variety of underlying processes. Additionally, OCTA captures only static flow at rest and may miss functional or dynamic vascular abnormalities. Our analysis focused on the superficial and deep plexuses, but changes in other vascular layers, such as the intermediate plexus or choriocapillaris, cannot be ruled out. Finally, while PROMs provide important insight into symptom burden, they are subjective and may not directly reflect underlying pathophysiology. Future multicenter, longitudinal studies with broader phenotyping are needed to validate and expand on these findings. In particular, a mechanistic link between neurodegeneration and OCT changes is needed to better interpret the results.

## 5 Conclusion

In this study, we investigated both structural and microvascular retinal changes in patients with PCS using retinal OCT imaging. While OCTA-derived vessel density in the superficial and deep vascular plexuses did not differ significantly from controls or correlate with symptom severity, OCT-based structural parameters such as pRNFL, GCIP, and TMV were significantly reduced in PCS patients and showed associations with validated symptom scores. These findings suggest that retinal structural alterations are associated with PCS and symptom severity and may be compatible with neuroinflammatory or neuroretinal changes. In contrast, the absence of OCTA abnormalities, particularly in a cohort with long post-infection intervals and mild acute disease, suggests that retinal blood flow at rest may be preserved, despite previously reported functional dysregulation in larger retinal vessels. Taken together, our results highlight the potential utility of OCT structural metrics for patient stratification and clinical monitoring in PCS, while underscoring the need for longitudinal, multicenter studies to further explore the temporal dynamics and mechanistic underpinnings of retinal changes in this complex condition.

## Consent to participate

Informed consent was obtained from all individual participants included in the study.

## Ethical approval

The study protocol received ethical approval from the Ethics Committee of the Technical University of Munich, School of Medicine, Klinikum rechts der Isar (Approval number: 2022-317-S-SR). This study was performed in accordance with the ethical standards as laid down in the 1964 Declaration of Helsinki and its later amendments or comparable ethical standards.

## Declaration of generative AI and AI-assisted technologies in the manuscript preparation process

During the preparation of this work, the authors used generative AI tools (Microsoft Copilot) to assist with language editing, restructuring, and clarification of phrasing. The authors critically reviewed and revised all generated content and take full responsibility for the accuracy and integrity of the final manuscript.

## Funding and conflicts of interest

Rebecca Wicklein received an intramural research grant from the Technical University of Munich, School of Medicine and was supported by the Deutsche Forschungsgemeinschaft (DFG, German Research Foundation) under Germany's Excellence Strategy within the framework of the Munich Cluster for Systems Neurology (EXC 2145 SyNergy – ID 390857198). Rebecca Wicklein received a poster award from Novartis. Martin J. Menten is funded by the German Research Foundation under projects 532139938. Timon Wallraven received an intramural research grant from the Technical University of Munich, School of Medicine.

Open Access funding enabled and organized by Projekt DEAL. This study is sponsored by the Bavarian State Ministry of Science and Art, as part of its Special Funding Program for the Scientific Coronavirus Research Projects of the Technical University of Munich School of Medicine at the University Medical Center “Rechts der Isar” (Funding Identification Number H.4001.1.7-53/7).

Furthermore, this study received Funding from the VADYS-ME Research Program.

## CRediT authorship contribution statement

**Michael Wunderle:** Data curation, Formal analysis, Visualization, Writing – original draft. **Rebecca Wicklein:** Investigation, Methodology. **Andrea Ribeiro:** Investigation, Writing – review & editing. **Javier Carbajo-Lozoya:** Writing – review & editing. **Anna Wöhnl:** Writing – review & editing. **Johanna Negele:** Investigation. **Veronika Kesseler:** Investigation. **Anna Wild:** Writing – review & editing. **Samuel Niedermayer:** Writing – review & editing. **Isabelle Lethen:** Writing – review & editing. **Maciej Lech:** Writing – review & editing. **Martin J. Menten:** Writing – review & editing. **Linus Kreitner:** Data curation. **Christoph Schmaderer:** Conceptualization, Project administration, Supervision, Writing – review & editing. **Timon Wallraven:** Conceptualization, Investigation, Methodology, Project administration, Writing – review & editing.

## Declaration of competing interest

The authors declare the following financial interests/personal relationships which may be considered as potential competing interests:

Martin Menten and Rebecca Wicklein report financial support was provided by German Research Foundation. Timon Wallraven reports financial support was provided by Technical University of Munich. Christoph Schmaderer reports financial support was provided by Bavarian State Ministry for Science and Art. Rebecca Wicklein received a poster award from Novartis If there are other authors, they declare that they have no known competing financial interests or personal relationships that could have appeared to influence the work reported in this paper.

## Data Availability

The datasets and analysis code generated and/or analyzed during the current study are available from the corresponding author on reasonable request.
