# Supplementary material for: Subclinical neurovascular and immune correlates of post-COVID-19 syndrome detected by retinal imaging
Source: Brain Behav Immun Health. 2026 Jul 25;56:101315. doi: 10.1016/j.bbih.2026.101315 (PMC13451791; doi:10.1016/j.bbih.2026.101315)
Supplement: Supplementary file 1 [file mmc1.docx]

# Supplementary Materials for

# Subclinical Neurovascular and Immune Correlates of Post-COVID-19 Syndrome Detected by Retinal Imaging

Michael Wunderle^1^, Rebecca Wicklein^2^, Andrea Ribeiro^1^, Javier Carbajo-Lozoya^1^, Anna Wöhnl^1^, Johanna Negele^1^, Veronika Kesseler^1^, Anna Wild^2^, Samuel Niedermayer^1^, Isabelle Lethen^1^, Maciej Lech^3^, Martin J. Menten^4,5^, Linus Kreitner^4^, Christoph Schmaderer*^1,6^, Timon Wallraven*^1^

^1^TUM School of Medicine and Health, Department of Nephrology, TUM University Hospital, Technical University of Munich, 81675 Munich, Germany

^2^TUM School of Medicine and Health, Department of Neurology, TUM University Hospital, Technical University of Munich, 81675 Munich, Germany

^3^Medizinische Klinik Und Poliklinik IV, LMU University Hospital Munich, Ziemssenstraße 5, 80336, Munich, Germany

^4^Chair for AI in Healthcare and Medicine, Technical University of Munich, Germany

^5^BioMedIA, Imperial College London, United Kingdom

^6^German Centre for Infection Research (DZIF), Munich Partner Site, Munich, Germany

*These authors contributed equally to this work

Addresses for correspondence:

Dr. Michael Wunderle^1^

Michael.Wunderle@mri.tum.de, Tel: +49 4140 5231, Fax: +49 4140 7734

Dr. Timon Wallraven^1^

Timon.Kuchler@mri.tum.de, Tel: +49 4140 5231, Fax: +49 4140 7734

To investigate whether retinal microvascular alterations in PCS patients are associated with subjective symptom burden, we performed correlation analyses between vessel density and patient-reported outcome measures (PROMs), including the PCS Severity Score, C19-YRS, PHQ9, and GAD7. While a weak negative trend was noted between the SVC and the C19-YRS as well as the PHQ9, none of the associations reached statistical significance (**Supplementary Figure 1**).

 **Supplementary Figure 1** Scatterplots show the correlation between vessel density parameters SVC (Panel **a**) and DVC (Panel **b**) and PROMs. Patient Health Questionnaire-9 (PHQ9), Generalized Anxiety Disorder-7 (GAD7), PCS Score and the C19-Yorkshire Rehabilitation Scale Symptom Severity (C19-YRS). Pearson or Spearman correlation coefficients (r) and associated p-values are shown.

To assess potential alterations of macular perfusion, we analyzed the foveal avascular zone (FAZ) area in PCS patients and controls. As shown in **Supplementary Figure 2a**, there were no significant differences in FAZ size between PCS patients and COVID-19 recovered individuals (p = 0.224), nor between PCS patients with and without ME/CFS (p = 0.167). Furthermore, FAZ area did not correlate significantly with any of the patient-reported outcome measures (PROMs), including PCS Score, C19-YRS, PHQ9, or GAD7 (**Supplementary Figure 2b**).

**Supplementary Figure 2** Comparison and correlation analyses of the foveal avascular zone (FAZ) area. **Panel a**: Boxplots show FAZ areas in patients with post-COVID syndrome (PCS) compared to recovered controls (left), and in PCS patients with and without comorbid ME/CFS (right). **Panel b**: Pearson or Spearman correlation analyses between FAZ area and patient-reported outcome measures (PROMs), including the PCS Score, C19-YRS, PHQ9, and GAD7. Shaded areas represent 95% confidence intervals. R and p values indicate Pearson or Spearman correlation coefficients.

A complete overview of retinal structural and microvascular parameters in PCS patients and COVID-19-recovered controls is provided in **Supplementary Table 1**.

| **Variable** | **COVID-19 recovered (mean ± SD)** | **PCS (mean ± SD)** | **p-value** |
| --- | --- | --- | --- |
| SVC (%) | 37.82 ± 1.79 | 36.00 ± 2.96 | **<0.001** |
| DVC (%) | 48.78 ± 2.02 | 48.35 ± 2.03 | 0.325 |
| pRNFL (µm) | 105.61 ± 9.64 | 101.95 ± 9.84 | **0.041** |
| RNFL (µm) | 33.49 ± 3.64 | 32.26 ± 3.29 | 0.061 |
| GCIP (µm) | 73.30 ± 4.00 | 71.15 ± 5.11 | **0.009** |
| INL (µm) | 34.97 ± 1.84 | 34.37 ± 1.94 | 0.086 |
| TMV (mm³) | 8.82 ± 0.30 | 8.62 ± 0.33 | **0.002** |

**Supplementary Table 1** Comparison of retinal structural and microvascular parameters between patients with PCS and COVID-19-recovered controls. Values are presented as mean ± standard deviation. P-values reflect unadjusted group comparisons.

To account for potential confounding by age and gender, multivariable regression analyses were performed for all key retinal parameters and are summarized in **Supplementary Table 2**.

| **Variable** | **OR** | **95% CI** | **p-value** |
| --- | --- | --- | --- |
| SVC (%) | 0.86 | 0.68 to 1.07 | 0.203 |
| DVC (%) | 1.10 | 0.84 to 1.44 | 0.495 |
| pRNFL (µm) | 0.97 | 0.93 to 1.01 | 0.185 |
| RNFL (µm) | 0.85 | 0.75 to 0.96 | **0.013** |
| GCIP (µm) | 0.92 | 0.84 to 1.00 | 0.058 |
| INL (µm) | 0.89 | 0.71 to 1.10 | 0.276 |
| TMV (mm³) | 0.10 | 0.02 to 0.42 | **0.005** |

**Supplementary Table 2** Multivariable linear regression analysis of retinal structural and microvascular parameters associated with PCS, adjusted for age and gender. Beta coefficients represent the estimated group effect for PCS compared with COVID-19-recovered controls. Confidence intervals (CI) are shown as 95% CI.

To assess the diagnostic potential of retinal parameters in distinguishing PCS patients from controls, we performed ROC curve analyses (**Supplementary Figure 3**). Logistic regression models combining GCIP with SVC **(a)**, TMV **(b)**, or pRNFL **(c)** demonstrated improved classification performance, suggesting that multimodal retinal biomarkers may enhance the identification of PCS-related changes.

**Supplementary Figure 3** Receiver operating characteristic (ROC) curve analysis of retinal parameters combined in logistic regression models adjusted for age and gender to predict PCS. Panel **a** ROC curve of a model combining SVC and GCIP thickness. Panel **b** ROC curve of a model combining GCIP thickness and TMV. Panel **c** ROC curve of a model combining GCIP and pRNFL thickness. The combined AUC is indicated in each panel.

As a sensitivity analysis addressing residual confounding due to age and sex differences, propensity score matching (1:1 nearest‑neighbor matching with a caliper of 0.4 based on age and gender) was applied. After matching, no significant differences were observed concerning demographic data and cardiovascular risk factors (**Supplementary Table 3**).

| **Variable** | **COVID-recovered n = 71** | **PCS n = 71** | **p-value** |
| --- | --- | --- | --- |
| **Age in years (median, IQR)** | 34.0 (28.0–45.5) | 36.0 (28.5–50.0) | 0.37 |
| **Gender, male (n (%))** | 21 (29.6%) | 16 (22.5%) | 0.44 |
| **BMI in kg/m^2^ (median, IQR)** | 23.0 (21.4–24.9) | 23.6 (20.6–25.8) | 0.99 |
| **Nicotine abuse (n (%))** | 9 (12.7%) | 7 (9.9%) | 0.79 |
| **Arterial hypertension (n (%))** | 5 (7.0%) | 10 (14.1%) | 0.27 |
| **Hypercholesterolemia (n (%))** | 17 (26.2%) | 28 (41.2%) | 0.100 |
| **Time since infection in days (mean ± SD)** | 549.5 (±359.7) | 444.3 (±241.5) | 0.047 |

**Supplementary Table 3** Sensitivity analysis using propensity score-matched cohorts. Unadjusted p-values reflect group comparisons using appropriate statistical tests.

In the matched cohorts, structural retinal parameters (GCIP thickness and TMV) showed nominally lower values in PCS patients compared with COVID-19-recovered controls, while OCTA-derived vessel density measures did not differ significantly (**Supplementary Table 4**). A similar pattern was observed in the subgroup analysis comparing PCS patients with and without comorbid ME/CFS (**Supplementary Table 5**), supporting the robustness of the primary findings and indicating that age imbalance is unlikely to fully explain the observed structural retinal differences.

| **Variable** | **COVID19-recovered n = 71** | **PCS n = 71** | **p** |
| --- | --- | --- | --- |
| **SVC (%)** | 37.70 ± 1.89 | 36.52 ± 2.82 | 0.059 |
| **DVC (%)** | 48.36 ± 2.22 | 48.31 ± 1.98 | 0.934 |
| **pRNFL (µm)** | 105.83 ± 8.57 | 103.76 ± 9.70 | 0.318 |
| **GCIP (µm)** | 73.53 ± 3.73 | 71.39 ± 5.17 | **0.032** |
| **TMV (mm³)** | 8.79 ± 0.30 | 8.61 ± 0.30 | **0.011** |

**Supplementary Table 4** Sensitivity analysis of OCT and OCTA parameters after propensity score matching. Data are presented as mean ± standard deviation (SD). Group comparisons between matched controls and PCS patients were performed using independent two-sample t-tests. SVC = superficial vascular complex; DVC = deep vascular complex; pRNFL = peripapillary retinal nerve fiber layer; GCIP = ganglion cell and inner plexiform layer; TMV = total macular volume. Vessel density was quantified separately for vessels >10 µm and <10 µm diameter as well as for the overall vascular plexus.

| **Variable** | **PCS without ME/CFS (mean ± SD)** | **PCS with ME/CFS (mean ± SD)** | **p** |
| --- | --- | --- | --- |
| **SVC (%)** | 37.32 ± 2.25 | 36.52 ± 2.88 | 0.230 |
| **DVC (%)** | 48.40 ± 2.07 | 48.25 ± 2.05 | 0.782 |
| **pRNFL (µm)** | 105.78 ± 10.19 | 103.05 ± 8.21 | 0.175 |
| **GCIP (µm)** | 73.31 ± 4.67 | 70.83 ± 4.69 | **0.017** |
| **TMV (mm³)** | 8.75 ± 0.30 | 8.58 ± 0.30 | **0.011** |

**Supplementary Table 5** Sensitivity analysis of retinal structural and microvascular OCT/OCTA parameters in propensity score-matched PCS patients with and without ME/CFS. Data are presented as mean ± standard deviation (SD). Group differences were assessed using independent two-sample Welch's t-tests. SVC = superficial vascular complex; DVC = deep vascular complex; pRNFL = peripapillary retinal nerve fiber layer; GCIP = ganglion cell and inner plexiform layer; TMV = total macular volume. Vessel density was quantified separately for vessels >10 µm and <10 µm diameter as well as for the overall vascular plexus.
